# Recombinant Thrombopoietin Effectively Shortens the Time to Response and Increases Platelet Counts in Elderly Patients with Severe Immune Thrombocytopenia

**DOI:** 10.3390/jcm11195763

**Published:** 2022-09-29

**Authors:** Yang Li, Lihua Sun, Feng Li, Ying Li, Yunhua Hou, Yahong Meng, Xiaohong Fan, Yunfeng Cheng, Fanli Hua

**Affiliations:** 1 Department of Hematology, Zhongshan Hospital, Qingpu Branch, Fudan University, 1158 Gong Yuan Dong Road, Shanghai 201700, China; 2 Department of Hematology, Zhongshan Hospital, Fudan University, 180 Fenglin Road, Shanghai 200032, China; 3 Center for Tumor Diagnosis & Therapy, Jinshan Hospital, Fudan University, Shanghai 201508, China; 4 Department of Hematology, Minhang Hospital, Fudan University, Shanghai 201199, China; 5 Institute of Clinical Science, Zhongshan Hospital, Fudan University, Shanghai 200032, China

**Keywords:** primary immune thrombocytopenia, recombinant human thrombopoietin, aged, treatment, autoimmune

## Abstract

Background: This study was conducted to investigate the short-term efficacy and safety of rhTPO for the management of severe ITP in the elderly as first-line treatment. Methods: A total of 54 elderly patients with severe ITP were studied, including 39 patients treated with a combination regimen of rhTPO plus standard treatment (glucocorticoid; rhTPO group) and 15 patients treated with glucocorticoid treatment alone (control group). The response rate, time to initial response, peak platelet counts, and time to peak platelet counts were compared, and clinical characteristics correlated with the efficacy of rhTPO were analyzed. The efficacy of rhTPO in the elderly is comparable to the non-elderly in terms of the OR, CR, time to initial response, and peak platelet counts. Results: There were no differences in the overall response (OR) and the complete response (CR) in the rhTPO group compared to the control group. The time to initial response in the rhTPO group was shorter than that in the control group (*p* = 0.032). In patients without intravenous immunoglobulin (IVIg) and platelet transfusion, the peak platelet counts in the rhTPO group were higher than those in the control group (*p* = 0.003). Conclusions: Standard glucocorticoid treatment plus rhTPO effectively shortens the time to response and increases platelet counts in the elderly with severe ITP.

## 1. Introduction

Primary immune thrombocytopenia (ITP) is an acquired autoimmune hemorrhagic disorder characterized by low platelet counts (PLT) and increased risks of bleeding [1,2]. The clinical manifestations of ITP range from asymptomatic to mild bruising, mucosal bleeding, and even intracranial hemorrhage. According to present ITP guidelines, corticosteroids remain the most important and effective frontline treatment of ITP, although accompanied by various side effects [3,4]. For life-threatening bleeding events, which are quite frequent in severe ITP, the major aim of management is to increase platelet counts to the safe level (usually defined as exceeding 30 × 10^9^/L) as soon as possible [5]. High-dose intravenous immunoglobulin (IVIg) and platelet transfusion are widely recommended as an emergency treatment to increase platelet counts rapidly [6].

Previous studies have established that antibody-mediated and/or T-cell-mediated platelet destruction plays a critical role in the pathogenesis of ITP [4,7,8,9,10]. Megakaryocytes are also strongly influenced (e.g., impaired development ability, diminished function of platelet release) [11]. Thrombopoietin (TPO) is an endogenous growth factor, being able to effectively promote the development of megakaryocytes and stimulate platelet production. Noticeably, the level of TPO in patients with ITP is usually relatively low, suggesting that TPO could be a potential target for the management of ITP. At present, TPO receptor agonists (TPO-RAs), such as eltrombopag and romiplostim, have shown remarkable efficacy in increasing platelet counts in patients with chronic/persistent ITP [12,13,14,15] and are thereby widely adopted as second-line treatment around the world. However, the administration of TPO-RAs in patients with severe ITP is still limited, partly due to its long response time; it usually takes 2–4 weeks to increase the platelet count to 50 × 10^9^/L in patients with ITP.

Recombinant human thrombopoietin (rhTPO) is a natural TPO analogue synthesized by genetic recombination technology and has been widely used for over 20 years in China. rhTPO is isolated and highly purified from Chinese hamster ovary (CHO) cells containing a gene for efficient expression of human thrombopoietin. As a glycosylated, full-length molecule, which is identical to endogenous TPO, the mechanism of rhTPO is more similar to that of natural TPO, and rhTPO shows profound effects on megakaryocyte development and platelet production. Previous studies have suggested that rhTPO markedly increases platelet counts in patients with chemotherapy-induced thrombocytopenia [16] as well as in patients with myelodysplastic syndromes and aplastic anemia [17,18]. Wang et al. conducted a multicenter randomized controlled trial in 2012, showing that platelet counts in patients with chronic ITP increase rapidly after rhTPO treatment [19]. Other studies have also demonstrated that rhTPO with/without glucocorticoid could both rapidly increase platelet counts and improve the complete response rate in severe newly diagnosed ITP [20], ITP with pregnancies [14,21], and corticosteroid-resistant/relapsed ITP [22]. Similarly, it is worth mentioning that a prospective, randomized, controlled clinical trial conducted by Hou suggested that the combination of high-dose dexamethasone with rhTPO could remarkably improve the initial and sustained response, strongly supporting the efficacy of rhTPO as a potential frontline treatment for newly diagnosed adult ITP [23].

Although extensive research has been carried out on the efficacy and safety of rhTPO in adult ITP, there is no single study that focuses on elderly patients with severe ITP. While ITP has been traditionally considered a disorder predominantly affecting young or middle-aged women, an epidemiological study conducted by Moulis et al. illustrated that the incidence of ITP in the elderly (>60 years old) was approximately 5–9/100,000 person-years, noticeably higher than 2.94/100,000 person-years in the non-elderly [24]. Additionally, it has been previously observed that the risk of bleeding in elderly patients, particularly in those with severe ITP (PLT<10 × 10^9^/L), is significantly higher than that in non-elderly patients, even at equivalent platelet counts [24,25,26]. Whereas corticosteroids are effective in elderly patients with ITP, the features of the elderly (e.g., various severe comorbidities, high bleeding risk) limit the long-term application of corticosteroids and make the side effects unacceptable. It is an urgent demand for elderly patients with severe ITP to find an alternative treatment that could increase platelet counts rapidly to reduce the risk of catastrophic bleeding events. However, there are no studies yet concentrating on the efficacy and safety of rhTPO in the elderly with severe ITP. It is still uncertain whether the elderly respond to rhTPO effectively as the non-elderly do. This retrospective study was designed to further clarify the efficacy of rhTPO as frontline treatment in elderly patients with severe ITP.

## 2. Methods

### 2.1. Patients

**Figure 1:**
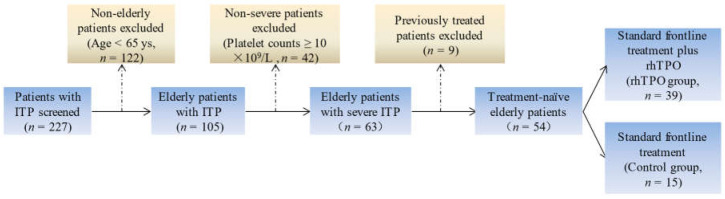
Flowchart of case selection.

In total, 227 patients with ITP between March 2016 and March 2021 were screened at the Department of Hematology of the Zhongshan Hospital Qingpu Branch, the Department of Hematology of Jinshan Hospital, and the Department of Hematology of Minhang Hospital. According to age, platelet counts, and clinical symptoms, 54 treatment-naive elderly patients (≥65 years) with severe ITP were enrolled in this study (Figure 1 shows the strategy of case selection). Of these 54 patients, 39 received standard frontline treatment (corticosteroids ± IVIg) plus rhTPO (rhTPO group) and 15 received standard frontline treatment alone (control group). All patients met the diagnostic criteria for severe ITP recommended by the updated international consensus report (PLT <10 × 10^9^/L and/or bleeding symptoms sufficient to mandate treatment) [1]. Bone marrow aspiration and examinations were applied to all patients to exclude secondary thrombocytopenia. Clinical data (including age, gender, comorbidities, bleeding symptoms, baseline platelet counts, megakaryocyte counts, lymphocyte subsets, quantitation of immunoglobulins, treatment regimen, platelet counts, and adverse events) were recorded for analysis. The bleeding manifestation was evaluated by an ITP bleeding scale recommended in a consensus of Chinese experts on the diagnosis and treatment of adult ITP by the Chinese Medical Association [5,20]. This scale showed strong assessment consistency and close correlation with the ITP-specific bleeding assessment tool (ITP-BAT), while having less time-consuming calculation. The baseline clinical characteristics of patients are shown in Table 1.

This study was approved by the Institutional Review Board of Zhongshan Hospital, Qingpu Branch, Fudan University, according to the Declaration of Helsinki. Written informed consent was obtained.

### 2.2. Treatments

Patients in the control group received standard frontline treatment (corticosteroids ± IVIg). Corticosteroid regimens included high-dose dexamethasone and methylprednisolone. High-dose dexamethasone was administered to patients at a dose of 40 mg/day for 4 days. Methylprednisolone was administered at a dose of 60~120 mg/day and then tapered off gradually. IVIg at a dose of 400 mg/kg/day for 5 days and/or platelet transfusion were prescribed to patients at the physician’s discretion. Patients in the rhTPO group received standard frontline treatment plus rhTPO. rhTPO was subcutaneously injected into patients at a dose of 15,000 U/day for no longer than 14 days and stopped when platelet counts exceeded 100 × 10^9^/L. The numbers of patients treated with IVIg and platelet transfusion are also shown in Table 1.

### 2.3. Outcome Evaluation

According to the criteria recommended by the international consensus [1], we defined response criteria based on the peak platelet counts in 14 days from initial treatment. The short-term response criteria were as follows: (1) complete response (CR), peak PLT ≥ 100 × 10^9^/L, without bleeding; (2) partial response (PR), peak PLT ≥ 30 × 10^9^/L but < 100 × 10^9^/L, without bleeding; and (3) no response (NR), peak PLT < 30 × 10^9^/L, or bleeding after treatment. The overall response (OR) was defined as CR plus PR. The time to initial response was defined as the duration from the day of initial treatment to the day when the platelet counts first exceeded 30 × 10^9^/L.

### 2.4. Statistical Analysis

GraphPad Prism 8.0.2 software was used for statistical analysis. Descriptive summaries of the data were performed in Excel (Microsoft Corp., Redmond, WA, USA). Normally distributed continuous variables are summarized as the mean ± SD, while non-normally distributed continuous variables are summarized as the median (first quartile, third quartile). Discrete variables are expressed as percentages. Quantitative and qualitative data were compared by the Mann–Whitney U and Fisher’s exact tests, respectively. The Pearson correlation analysis method was used to analyze the correlation between two groups. Statistical significance was defined as *p* < 0.05 and high significance as *p* < 0.001.

## 3. Results

### 3.1. Response Rate

Overall, no significant difference in the response rate between the rhTPO group and the control group was found. The overall response rate in the rhTPO group was 100% (39/39), slightly higher than 93.3% (14/15) in the control group (*p* = 0.278). The complete response rates were 71.8% (28/39) in the rhTPO group and 73.3% (11/15) in the control group (*p >* 0.999). As high-dose IVIg and platelet transfusion could rapidly increase the platelet counts of patients with severe ITP, patients treated with IVIg and platelet transfusion were excluded and the rest were analyzed. The overall response rates in the rhTPO group (17 cases) and the control group (7 cases) were 100% (17/17) and 85.7% (6/7), respectively (*p* = 0.292). The complete response rate in the rhTPO group was remarkably higher than that in the control group (82.4% (14/17) vs. 42.9% (3/7)), while showing no statistical significance (*p* = 0.137), as shown in Table 2.

### 3.2. Time to Initial Response

The time to initial response was defined as the duration from the day of initial treatment to the day when platelet counts first exceeded 30 × 10^9^/L. Within 14 days after initial treatment, the time to initial response in the rhTPO group was 5.0 (3.0, 6.0) days, significantly shorter than 6.0 (4.0, 7.0) days in the control group (*p* = 0.032). Among patients not receiving IVIg and platelet transfusion, the figure for the rhTPO group was still shorter than that for the control group (4.0 (3.0, 6.0) days vs. 7.0 (4.0, 10.0) days, *p* = 0.041; Table 2).

### 3.3. Peak Platelet Counts

The peak platelet counts in the rhTPO group were 141.0 (91.0, 253.0) ×10^9^/L, higher than 127.0 (84.0, 209.0) × 10^9^/L in the control group, with no statistical significance (*p* = 0.276). However, after excluding patients receiving IVIg and platelet transfusion, the peak platelet counts in the rhTPO group were markedly higher than those in the control group (159.0 (114.5, 263.0) × 10^9^/L vs. 84.0 (46.0, 104.0) × 10^9^/L, *p* = 0.003). In terms of the time to peak platelet counts, there was no significant difference between the rhTPO (8.3 ± 2.9 days) and control (8.8 ± 1.7 days; *p* = 0.544) groups. Similarly, in patients not treated with IVIg and platelet transfusion, the time to peak platelet counts in the rhTPO and control groups were 7.8 ± 3.2 days and 8.7 ± 2.4 days, respectively, with no statistical significance (*p* = 0.487); see Table 2. 

### 3.4. Factors Related to the Time to Initial Response

There was no significant correlation between age and the time to initial response (R2 = 0.011, *p* = 0.529). Differences of gender also had no significant effect on the time to initial response (t = 1.708, *p* = 0.096). Other clinical characteristics collected in this study (e.g., the megakaryocyte counts of bone marrow, the total lymphocyte counts, and the level of immunoglobulin) did not show any correlation with the time to initial response as well.

### 3.5. Adverse Events

Current treatments were well tolerated. Only one case (6.7%) of infection during hospitalization in the control group was observed, while there were no infection cases (0%) in the rhTPO group (*p* = 0.278). No catastrophic bleeding events were observed during hospitalization. While there were seven cases (18.0%) in the rhTPO group and one case (6.7%) in the control group where the peak platelet counts exceeded the upper normal limit (>300 × 10^9^/L), no thromboembolic events were reported during hospitalization.

### 3.6. Efficacy Comparison with Non-Elderly Patients

A total of 35 rhTPO-treated non-elderly patients (<65 years) with severe ITP were selected from the same database. The screening criteria were the same as those for elderly patients, except for age. The baseline platelet counts were 3.0 (1.0, 8.0) × 10^9^/L in the elderly and 5.0 (3.0, 7.0) × 10^9^/L in the non-elderly, respectively (*p* = 0.213). The overall response rates in the elderly and non-elderly were both 100% (*p* > 0.999). The complete response rate in the elderly was 71.8% (28/39), slightly lower than 82.9% (29/35) in the non-elderly, with no significant difference (*p* = 0.284). There was also no significant difference in the time to initial response between the two groups. The figure for the elderly was 5.0 (3.0, 6.0) days, consistent with 5.0 (4.0, 5.0) days for the non-elderly (*p* = 0.919). Similarly, the peak platelet counts in the non-elderly were markedly higher than those in the elderly (199.0 (125.0, 384.0) × 10^9^/L vs. 141.0 (91.0, 253.0) × 10^9^/L), while showing no statistical significance (*p* = 0.115). The time to peak platelet counts showed the same trend, with 8.3 ± 2.9 days in the elderly and 8.4 ± 2.5 days in the non-elderly (*p* = 0.851), as shown in Table 3.

## 4. Discussion

The urgent and optimum therapeutic goal for elderly patients with severe ITP is to increase platelet counts to the safe level and therefore avoid catastrophic bleeding events. Immune abnormalities lead to increased platelet destruction and decreased platelet production in patients with ITP [11,27,28,29]. Given the high risk of bleeding in elderly patients, it is necessary to find a better treatment option for elderly patients with severe ITP. Recombinant human TPO is a full-length and glycosylated TPO expressed in Chinese hamster ovary cells and purified by bioengineering techniques. It was approved by the China State Food and Drug Administration as a second-line treatment option for ITP. As illustrated in the Introduction section, there are no studies concerning the efficacy of rhTPO in elderly patients with severe ITP. This is the first study to focus on elderly patients with severe ITP that analyzed the short-term efficacy of rhTPO as frontline treatment. Elderly patients have a high risk of bleeding, and the risk of fatal intracerebral hemorrhage is much higher in elderly patients than that in young patients if the platelet count is not promptly and rapidly increased. As first-line prothrombogenic therapy, TPO-RAs or TPO can be helpful for rapid platelet increase in elderly patients with ITP [15]. This study provided clinical evidence for prothrombogenic therapy to become first-line treatment.

In our study, the most important clinical finding was that the time to initial response in the rhTPO group was significantly shorter than that in the control group, with a considerable difference of 1 day. This result could be still observed after excluding patients treated with IVIg and platelet transfusion, suggesting that rhTPO plays a crucial role in accelerating the increase in platelet counts, being independent of IVIg and/or platelet transfusion. Given the higher risk of bleeding and ITP-related death in the elderly compared to the non-elderly [6], it is the primary aim to increase the platelet count to the safe level as quickly as possible. Our results showed that the time to initial response reduced by 1 day in the rhTPO group, demonstrating that standard frontline therapy plus rhTPO could improve the platelet counts to the safe level more quickly and effectively in elderly patients with severe ITP, thereby potentially reducing the incidence of catastrophic bleeding episodes.

In our study, there was no occurrence of anti-TPO in patients, which might be related to the short-time use of TPO and the relatively low incidence of anti-TPO in the Chinese population. The response rate of the elderly to rhTPO treatment was also evaluated. The data showed that the OR in the rhTPO group was slightly higher than that in the control group, whether or not excluding IVIg and platelet transfusion. The CR in the rhTPO group was consistent with that in the control group, and it became remarkably higher than that in the control group after excluding patients treated with IVIg and platelet transfusion. However, the differences in the OR/CR rates between the two groups did not reach statistical significance. Previous studies have reported that rhTPO combined with glucocorticoids could significantly improve the complete response rate in patients with severe ITP [19]. The limited sample size in our study possibly led to a different conclusion. Well-designed prospective, randomized controlled studies are necessary to clarify the role of rhTPO as frontline therapy in improving the response rate in elderly patients with severe ITP.

In terms of the peak platelet counts and the time to peak platelet counts, this study did not show any significant difference between the two groups. However, in the subgroup of patients not receiving IVIg and platelet transfusion, the peak platelet counts in the rhTPO group were significantly higher than those in the control group. Considering the sample size of this subgroup (only 24 cases), the result needs to be confirmed by further studies.

Interestingly, there was a result that seems to be contrary to that of previous studies, which have suggested that TPO-RAs are associated with a higher risk of thromboembolic events [30,31], especially in patients with other risk factors for thrombosis (e.g., age over 65 years). In this study, there were several patients whose peak platelet counts exceeded the upper normal limit (>300 × 10^9^/L), but no thromboembolic events were reported during hospitalization. It seems that rhTPO may not greatly increase the risk of thrombosis in elderly patients with severe ITP. However, clinicians should still pay attention to elderly patients treated with rhTPO, because rapidly increased platelet counts may still put elderly patients at an extremely high risk of thromboembolic events.

rhTPO was well tolerated in the elderly. Adverse events were mild, and there were no catastrophic bleeding events as well as thromboembolic events reported during hospitalization, while one case of infection occurred in the control group.

To further explore the difference in the efficacy of rhTPO treatment between elderly and non-elderly patients, non-elderly patients with severe ITP (also treated with rhTPO) were selected from the same database and the data were analyzed. The results showed that there was no significant difference in all targets between the two groups, suggesting that the efficacy of rhTPO in elderly patients is comparable to that in non-elderly patients. As it is usually thought that the response of the elderly to rhTPO could be poorer than that of the non-elderly, this result probably suggests that rhTPO is also an effective treatment in elderly patients with severe ITP.

In conclusion, rhTPO combined with standard frontline treatment could significantly shorten the time to initial response in elderly patients with severe ITP and could increase platelet counts to the safe level rapidly, thereby potentially reducing the risk of bleeding. In addition, after excluding the influence of IVIg and platelet transfusion, rhTPO significantly improves peak platelet counts without eliciting extra thromboembolic events. Given the retrospective nature and small sample size of this study, well-designed prospective studies with larger sample sizes and longer follow-up periods are needed to verify the efficacy and safety of rhTPO as the frontline option in elderly patients with severe ITP.

## Figures and Tables

**Table 1:** Baseline clinical characteristics.

Characteristics	rhTPO Group (*n* = 39)	Control Group (*n* = 15)	*p*-Value
Age (years old)	71.4 ± 8.9	75.0 ± 7.0	0.169
Gender (male/female)	14/25	10/5	0.066
Baseline platelet (×10^9^/L)	3.0 (1.0, 8.0)	3.0 (2.0, 7.0)	0.861
Bleeding score	3.3 ± 2.1	4.2 ± 2.5	0.265
Number of IVIg (%)	33.3 (13/39)	26.7 (4/15)	0.751
Number of platelet transfusions (%)	38.5 (15/39)	40.0 (6/15)	>0.999

Abbreviations: rhTPO, recombinant human thrombopoietin; IVIg, intravenous immunoglobulin.

**Table 2:** Short-term efficacy of initial treatment with rhTPO in elderly patients with severe ITP.

	Total		*p*-Value	Without IVIg and Platelet Transfusion		*p*-Value
	rhTPO (*n* = 39)	Control (*n* =1 5)		rhTPO (*n* = 17)	Control (*n* = 7)	
Overall response rate (%)	100.0 (39/39)	93.3 (14/15)	0.278	100.0 (17/17)	85.7 (6/7)	0.292
Complete response rate (%)	71.8 (28/39)	73.3 (11/15)	>0.999	82.4 (14/17)	42.9 (3/7)	0.137
Time to initial response (days)	5.0 (3.0, 6.0)	6.0 (4.0, 7.0)	0.032	4.0 (3.0, 6.0)	7.0 (4.0, 10.0)	0.041
Peak platelet counts (×10^9^/L)	141.0 (91.0, 253.0)	127.0 (84.0, 209.0)	0.276	159.0 (114.5, 263.0)	84.0 (46.0, 104.0)	0.003
Time to peak platelet counts (days)	8.3±2.9	8.8±1.7	0.544	7.8±3.2	8.7±2.4	0.487

Abbreviations: ITP, immune thrombocytopenia; rhTPO, recombinant human thrombopoietin; IVIg, intravenous immunoglobulin.

**Table 3:** Comparison of short-term efficacy in elderly and non-elderly patients.

	Elderly (*n* = 39)	Non-Elderly (*n* = 35)	*p*-Value
Overall response rate (%)	100.0 (39/39)	100.0 (35/35)	>0.999
Complete response rate (%)	71.8 (28/39)	82.9 (29/35)	0.284
Time to initial response (days)	5.0 (3.0, 6.0)	5.0 (4.0, 5.0)	0.919
Baseline platelet counts (×10^9^/L)	3.0 (1.0, 8.0)	5.0 (3.0, 7.0)	0.213
Peak platelet counts (×10^9^/L)	141.0 (91.0, 253.0)	199.0 (125.0, 384.0)	0.115
Time to peak platelet counts (days)	8.3 ± 2.9	8.4 ± 2.5	0.851

## Data Availability

Y.L. had access to the complete dataset used in the study and takes responsibility for the integrity of the data and accuracy of the data analyses. The dataset is available upon justified request.
